# June

**Published:** 1890-06-14

**Authors:** 


### JUNE.

That compendium of universal knowledge, "Whitaker's Almanac," informs us that on June 1st notices are to be given to those who have not paid all their poor rates and taxes, perhaps with the view of ulterior proceedings against such recreants. But it is not of rates and taxes and unpleasant matters we ought to think in early June, for June is the first month in which our summer really asserts its supremacy. Much of the preceding month of May is often more or less under the subjection of snarling east winds, which render the temperature more disagreeable and dangerous than the persistent cold experienced in mid-winter, especially when, as so frequently is the case, what should be April showers persist in falling in May. For such reasons the prudent, who desire to avoid chill and its consequences, would do well to follow the old advice, "Until the month of May is out, ne'er change a clout." But June having commenced, we may with confidence don cooler clothing. The Romans welcomed the early part of the month with suitable fêtes in honour of vestals, fire, and fecundity, for the month of June was considered *nuptiis aptissimus*. And some of the old old customs have survived in one shape or other even to the present day. In the old Latin calendar June was the fourth month. At the period of the Julian reform of the calendar its days were only twenty-nine, and to these Cæsar added a thirtieth, which we still retain. Ovid on his "Fasti" makes Juno assert that the name of the month was expressly given in her honour: an account of the origin of the name which we may accept with as good a grace as we may entertain any other which has been offered. Our Anglo-Saxon forefathers called it "the dry moanth," "the sear moanth," "the midsummer moanth," "the earlier mild moanth," the "litha ærra." Let us hope the month of June, 1890, will prove worthy of the ancient reputation of the season. For June is the season when summer having entered into undisturbed possession, the holiday-tide for tourists and travellers may be said to have commenced. "The rich and varied herbage of the fields," the song of birds, the blaze of insect-life so suddenly at its maximum, the weather fine but not sultry, as in July and August, are characteristics of the country in June, and tempt dwellers in towns to rural delights. It has been said that most men are like Horace. They admire rural life—but for all that they remain with the modern representative of Augustus. Nevertheless, it is generally admitted that the first week in the country is rapture; although the second may only afford calm content, while the third excites utter weariness and desire to be gone. There is no accounting for tastes. Therefore, let those who tire of "sitting in the sunshine warm, sheltered from the vagrant breeze;" or those who, in the luxury of the stillness and repose of forest, moor, mountain, and dale, become homesick for the London streets, and the ceaseless noise of traffic—let all such persons limit their country visits to the period of rapture. Even London itself is seen to best advantage on a bright day in June. The trees in the squares and parks and the grass are at the greenest, for the bright cheery aspect of nature has penetrated even thus far; smoke and fog is at the minimum, and heat has not attained the maximum of July and August. But no one should miss Richmond in June, with its view over the sea of trees below, and the serpentine river flowing through Twickenham meadows. The neighbourhood of Hampstead Heath is also a fine place for a June stroll, while on the other sides of London there are Greenwich Park, Shooters Hill, Streatham Common, Harrow, Windsor Forest, and Virginia Water. For those desiring to go further, Tunbridge Wells is a pleasant place to stop at, when June assumes its mantle of dark green. Those appreciating rural scenery cannot fail to enjoy the varied surface passed over, when moving round the neighbourhood of Tunbridge Wells. But "down Devon" is perhaps better, although so different. A lane in Devonshire is a perfect snuggery and study. The road is shaded by trees, and the banks are carpeted with flowers, among which the pink of the "herb Robert," the bright blue of the "bird's eye," and the yellow spikes of the "navelwort," are conspicuous, while the hawthorn blossoms are still on the hedge, and the thrush sings.

June 2nd is the anniversary of two great naval victories—that by the Duke of Albermarle over Van Tromp the Dutchman in 1666; and that by Lord Howe over the French fleet in 1794. June 2nd is also the anniversary of the death of a once public notoriety "Peit, the miser," who departed this life in 1803. He made a rash vow that when he had accumulated one thousand pounds he would treat himself to a pint of stout every day. Unfortunately for Peit an additional tax was placed on malt exactly at the time when the first thousand was counted. Whereupon Peit broke his vow, contenting himself with half-a-pint, and revenging himself by composing the following lines:

> They raise the price of table drink,
> What is the reason do you think?
> The tax on malt, the cause I hear,
> But what has that to do with beer?

Old Stowe says, on June 16th "King John, hee there appointed to meete with ye barons in ye meddowe betwixt Staines and Winsoore, which appointment hee observed, and then granted the liberties without anie difficultie," so June 16th should always be a memorable day in English history. But as in 1752 our calendar was reformed by the omission of thirteen days, the present June 16th is not the real anniversary of the day when "he did meete with ye barons in ye meddowe." But the most important day of the month is the 26th—Midsummer Day. Then, although our earth is really a greater distance away from the sun than in spring, the sun's rays descend more vertically, and therefore with greater force. The days in Europe reach their greatest length, and the "land of the midnight sun" knows no night. The northern part of the temperate zone has now a genial warmth, while the southern part, becoming tropical, is deserted by the winter visitors.

In former times Midsummer Eve was a counterpart of Christmas Eve, and passed in either feasting or fasting, according to the ideas and superstitions of the multitude. Among curious customs which prevailed may be mentioned leaping over a bonfire. Dr. Hicks states: "A bonfire is a festive or triumphal fire; it is a Ba-al or Boel-fyr, and hence by legitimate change of letters, bœn-fire, or, as we now term it, bon-fire." General Forlong, in his "Rivers of Life," tells us that at the midsummer period people were to cleanse themselves morally by leaping over these fires, running through them, or by swimming in water. Another curious custom was gathering fern seeds, which were presumed to possess magical power if procured on Midsummer Eve. Carrying the image of a flying dragon or idol about is an ancient midsummer custom, supposed to have been derived from the gorgeous East, where the passions of dragons were deemed to be excited to the maximum by the intense heat of the season, and therefore service and propitiation were rendered by female bearers.

In an enumeration of notable anniversaries occurring in June, the coronation of Queen Victoria on June 28th, 1838, must not be omitted. An old song describes how a countryman, having heard the village barber read the news, journeyed to London to witness the ceremony. This he achieved, but then found he had "paid dearly for his fancy." He "lost a sovereign and his purse, and on examination, his watch, which never went before, had gone at coronation. Although he lost his money by some thieves his pocket fumbling, you must not suppose that ever he did give himself to grumbling. He liked the sight so well that he, without any hesitation, would lose another sovereign for another coronation."

